# Model-Based Analysis of Flow-Mediated Dilation and Intima-Media Thickness

**DOI:** 10.1155/2008/738545

**Published:** 2009-04-06

**Authors:** G. Bartoli, G. Menegaz, M. Lisi, G. Di Stolfo, S. Dragoni, T. Gori

**Affiliations:** ^1^Department of Information Engineering, University of Siena, 53100 Siena, Italy; ^2^Department of Computer Science, University of Verona, 37134 Verona, Italy; ^3^Department of Internal, Cardiovascular, and Geriatric Medicine, University of Siena, 53100 Siena, Italy; ^4^Medizinische Klinik für Kardiologie und Angiologie, 55131 Mainz, Germany

## Abstract

We present an end-to-end system for the automatic measurement of flow-mediated dilation (FMD) and intima-media thickness (IMT) for the assessment of the arterial function. The video sequences are acquired from a B-mode echographic scanner. A spline model (deformable template) is fitted to the data to detect the artery boundaries and track them all along the video sequence. The a priori knowledge about the image features and its content is exploited. Preprocessing is performed to improve both the visual quality of video frames for visual inspection and the performance of the segmentation algorithm without affecting the accuracy of the measurements. The system allows real-time processing as well as a high level of interactivity with the user. This is obtained by a graphical user interface (GUI) enabling the cardiologist to supervise the whole process and to eventually reset the contour extraction at any point in time. The system was validated and the accuracy, reproducibility, and repeatability of the measurements were assessed with extensive *in vivo* experiments. Jointly with the user friendliness, low cost, and robustness, this makes the system suitable for both research and daily clinical use.

## 1. INTRODUCTION

The assessment and characterization of the endothelial function (i.e., the production of protective factors from the vascular endothelium) is a topic of both clinical and research importance. Techniques that assess this function have been proposed as useful tools in the diagnosis and management of cardiovascular diseases [1]. In particular, endothelium-dependent changes in hemodynamics (blood flow, blood pressure, vascular diameter, and tone) have been used as surrogate markers of vascular health and in the management of patients with cardiovascular diseases [2–4]. In detail, the endothelium responds to changes in intravascular shear stress by releasing several compounds which determine relaxation of smooth muscle cells and, subsequently, vasodilation [5]. In humans in vivo, such changes in shear stress can be experimentally determined by inflating to suprasystolic pressure a pneumatic cuff around the forearm for 4 minutes and 30 seconds. Upon release of the cuff, the sudden increase in blood flow (and shear stress) that follows reperfusion is a potent stimulus for endothelium-dependent vasorelaxation, which can be observed using ultrasounds [6, 7]. In sum, the endothelium shows measurable responses to flow changes, determining endothelium dependent, flow-mediated dilation (FMD). These measures might have a clinical potential, as several studies show an association between impaired FMD and poorer prognosis [2–4].

High-resolution B-mode ultrasonography (US) is a cheap and noninvasive technique that permits depiction of peripheral arteries. Image analysis techniques allow accurate, objective, and repeatable measurement of the diameter of such arteries. Several methods based on the detection of the edges of the arterial wall have been proposed over the last ten years. The first studies used a tedious manual procedure, which had a high intra- and interobserver variability. Some interactive methods tried to reduce this variability by attracting manually drawn contours to image features, like the maximum image gradient, where the vessel bound is assumed to be localized. Some more recent efforts are focused on dynamic programming or deformable models and neural networks [8–15].

All these methods present some common limitations. First, edge detection techniques are often undermined by speckle noise. Second, most methods require expert intervention to manually guide or correct the measurements, thus being prone to introduce operator-dependent variability. As well, temporal continuity of the measurements (as compared to measurements at predetermined time points) is another aspect that has not been exploited enough in previous work. Finally, there is a general lack of large-scale validation studies in most of these techniques.

However, getting rid of the first two factors out of the scopes of this contribution as, in general, it is not desired by cardiologists. The presence of speckle noise cannot be avoided unless denoising is performed on the images, which could alter the diagnostically relevant information. Furthermore, the doctor must be enabled to intervene in the segmentation process in case some causes of degradation (like a sudden movement of the patient) make the algorithm diverge.

Accordingly, in the present contribution we propose a new technique for the detection of artery boundaries which has the advantages of (i) improved accuracy and robustness to noise in the contour identification due to the use of a spline-based model for the artery contours; (ii) improved human-machine interaction through the design of an ad hoc graphical user interface (GUI); (iii) low-computational complexity; (iv) portability, and (v) low cost. In this respect, it is worth mentioning that currently there is no commercially available system able to perform both the FMD and IMT measurements automatically. Furthermore, the solution proposed in [11] for assessing the FMD functionality is proprietary and expensive, besides not being portable since it is implemented on device.

This paper is organized as follows. Section 2 describes the proposed system, including the experimental setup, preprocessing, and contour extraction. Section 3 illustrates the system validation from the clinical point of view as well as the performance, and Section 4 derives conclusions. Finally, the GUI is illustrated in the appendix.

## 2. METHODS

### 2.1. Experimental setup

The technique that was used for measuring the FMD is described in detail in [16–18]. Briefly, the left arm is immobilized using a deflation pillow and a pneumatic cuff is placed at the wrist (i.e., distally to the imaged site). The radial artery is imaged 10–15 cm below the elbow at rest for 60 seconds to acquire the *baseline* diameter. A pneumatic cuff positioned around the wrist is then inflated to 250 mmHg. After an interval of 4′30′′, the cuff is deflated to achieve reactive hyperemia. The artery is imaged for the following 4′30′′. Studies have shown that FMD does not change for occlusion times comprised between 4′30′′ and 10′ [19]. ECG-triggered end-diastolic frames are captured at a frame rate of 1 second by the Acuson Sequoia 512 high-resolution echograph from Sonoma Health, Calif, USA (http://www.sonomahealth.com/).

Each FMD movie consists of 600 frames: 60 for the baseline period, 270 during cuff inflation, and 270 after cuff deflation. FMD is defined as 100 times (maximum diameter after cuff deflation minus baseline diameter) divided by the baseline diameter. FMD is expressed as percentage increase from this resting diameter.

For intima-media thickness (IMT) analysis, an image of the posterior common carotid wall 1 cm proximal to the aortic bulb was taken using a linear 15 MHz probe and an Acuson Sequoia 512 ultrasound equipment. IMT was calculated as the mean distance between the two spline guides positioned at the *lumen-intima* and the *media-adventitia* interfaces (“leading edge” principle) [20].

### 2.2. Model-based segmentation of the vessels boundaries

The vessel segmentation consists in modeling the artery boundaries by a couple of cubic splines which are independently fitted to the contours following the minimization of a cost function. Preprocessing on the echographic images is performed in order to (i) improve the visual quality of the frames for visual inspection; (ii) support the segmentation algorithm; (iii) speed up the processing to enable real-time functionality. However, this does not affect the accuracy of the segmentation, as will be discussed in what follows.

### 2.3. Preprocessing

Data were acquired by a workstation and put in AVI format by a freeware software tool (VirtualDub).

**Figure 1:**
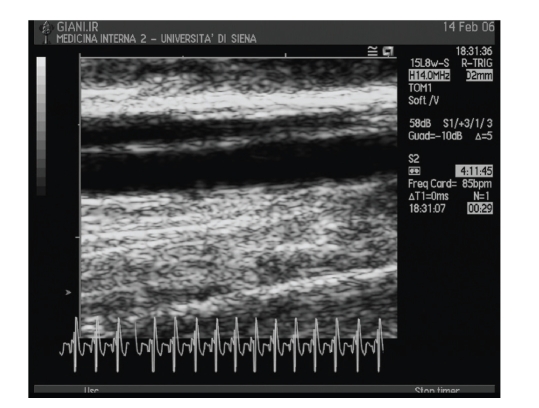
Typical FMD image resulting from the echo scanner.

Figure 1 shows a typical image. In order to reduce the required memory storage and to speed up the subsequent processing, the user can choose to low-pass filter and downsample the frames by a factor two along both dimensions. This reduction in size does not compromise the accuracy of the measurement of the vessel diameter. This was proved by comparing the output parameters (FMD, diameters, and IMT) obtained by processing some test videos with and without subsampling and calculating the impact on accuracy. Results show that this introduces a variability of about 0.1–0.3% in the FMD measure, which is negligible with respect to the other sources of variability (like the patient movement during the acquisition) and it is of the same order of magnitude of others (the positioning of the observation window and the setting of the metric units). However, we would like to emphasize that subsampling is an option and can be switched off. The results presented here were obtained using original size full images.

Two preprocessing steps are performed before boundary detection: contrast enhancement by histogram stretching and sharpening via a negative of Laplacian filter of size 3 × 3. Such operations are functional to both visual inspection and boundary detection.

In order to further speed up the processing, the graphical interface enables the selection of a region of interest (ROI). Again, this is based on the fact that cardiologists identify a region in the image which corresponds to the portion of the vessel that is suitable for the measurement under way. However, the option can be disabled and the analysis can be performed on the entire images. The selection of the ROI is performed on a representative image obtained by averaging of all the images along the sequence. In this way, the positioning of the observation window in an artifact-free region is ensured. The window is then propagated along the sequence.

**Figure 2:**
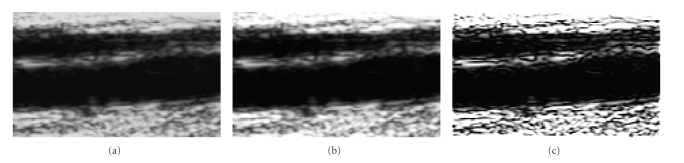
Preprocessing. (a) Original image; (b) after histogram stretching; (c) after sharpening.

The effects of the preprocessing steps are illustrated in Figure 2. The original cropped image is shown in Figure 2(a), the effect of contrast enhancement through histogram stretching is illustrated in Figure 2(b), and the final image after sharpening is shown in Figure 2(c). As mentioned above, these steps support the subsequent contour extraction process as they enhance the contrast without compromising the accuracy of the target measurement, as discussed above.

### 2.4. Vessel segmentation

The a priori knowledge about the shape of the vessels is exploited for segmentation. It is a fact that the vessel contours are smooth, have very small curvature, and are almost perfectly horizontal. Accordingly, they can be conveniently modeled by low-order polynomials. The use of a cubic spline proved to be suitable to the purpose. Importantly, as mentioned above, local tissue modifications corresponding to high curvatures must be discarded for the analysis as they would produce spurious changes in the estimated diameter that do not hold any information relevant to the measurement of the endothelial function. The choice of using smooth functions to model the contour of the vessel solves this problem as local high curvature segments are automatically disregarded. Should a more complex border be identified, it would be sufficient to add more knots in the interpolation procedure. We refer to [21, 22] for a more detailed discussion about spline-based interpolation.

Two cubic splines are independently fitted to the artery walls and progressively propagated along the video sequence such that the final estimation of the contours in image *n* is used for initialization in image *n* + 1.

The spline interpolating curve *S*(*x*) is a piecewise continuous function consisting of a set of polynomial segments *S*_*k*_(*x*). Each segment can be written as a polynomial of third degree:

(1) $$S_{k}(x)=\overset{3}{\underset{i=0}{\sum }}\;a_{k}^{i}{\left(x-x_{k}\right)}^{i},\;\forall x\in [x_{k},x_{k+1}].$$

The set of points {*x*_*k*_, *y*_*k*_, *k* = 1,…, *n*} is the control points of the curve (the *knots*) which are set manually on the first image of the video sequence for initialization. Results (that are not reported here) show that three to five points allow capturing the shape of the artery wall with the desired accuracy. Due to the smoothness of the contours, the knots were equally spaced along the horizontal dimension (i.e., the {*x*_*k*_} coordinates were kept unchanged) while the vertical positions {*y*_*k*_} of the knots are the free parameters. The value of the {*a*_*k*_^*i*^} coefficients follow.

**Figure 3:**
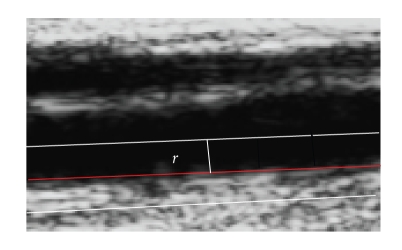
Search region for positioning the spline curve.

The objective of the cost function is the maximization of the contrast between the image regions that are above and below each curve, respectively, within a predefined radius, as illustrated in Figure 3. 

Let *r* be the radius of the circular-section tube centered on the current estimation of the boundary. The radius was set to either 12 or 6 pixels in case the downsampling option is switched off and on, respectively. Let then Ω_*u*_ and Ω_*l*_ be the two regions intercepted by the tube above and below the spline curve. The average gray values of these regions are

(2) $${\overset{¯}{g}}_{\nu }=\frac{1}{\left|\Omega_{\nu }\right|}\underset{\Omega_{\nu }}{\sum }\;I(i,j),$$

where *I*(*i*, *j*) is the gray level at position (*i*, *j*) in the image, |Ω_*ν*_| is the cardinality of the set, and *ν* = *u*, *l*, where *u* stands for *upper* and *v* for *lower*, respectively. The cost function *f* is defined as the difference of the average gray levels in the two regions, that is, the measure of the contrast that we use here:

(3) $$f=-|{\overset{¯}{g}}_{u}-{\overset{¯}{g}}_{l}|.$$

The average intensity is calculated columnwise such that

(4) f=−∑j=1Ncol|g¯u(j)−g¯l(j)|,g¯u(j)=∑i=y^jy^j+r I(i,j),g¯l(j)=∑i=y^j−ry^j I(i,j)

with ${\hat{y}}_{j}$ being the estimation of the vertical coordinate of the spline point at horizontal position *j* at the current iteration. A multidimensional unconstrained nonlinear minimization procedure is used to determine the y^k,opt optimal values for the *y* coordinates of the splines knots:

(5) y^k,opt=miny^k{f}.

**Figure 4:**
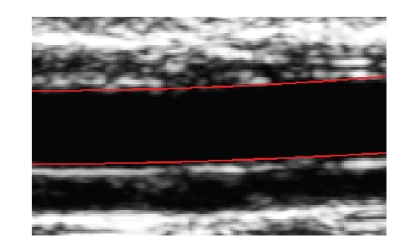
Results: the modeling splines corresponding to the upper and lower boundaries are represented as red curves.

Given the definition of the cost function, it is straightforward to conclude that contrast enhancement improves the performance. An example of the result is shown in Figure 4, where the model is superimposed to the ROI image. To reach the segmentation of the entire set of images, the model is propagated through the sequence such that the boundaries that have been determined in frame *n* serve as initialization of the search procedure in frame *n* + 1. However, the GUI allows the user to supervise the process and eventually stop it at any time in case of unsatisfactory result. In this case, the spline knots can be repositioned manually for the remaining of the analysis. Alternatively, the user can activate a temporal filtering operation to eliminate badly estimated contours in one or a group of frames and replace them with an a posteriori prediction. The cardiologist can then decide about the suitability of the result and thus decide to repeat the measurement or to drop the corresponding set of frames.

### 2.5. Diameter estimation

Once the models are fitted to the boundaries, the artery diameter is estimated as the average of the columnwise difference among the vertical coordinates of the two splines over the frame (the subscript *opt* was omitted for simplicity of notations):

(6) dm=1Ncol∑j=1Ncol(y^j,u−y^j,l),

where *m* is the frame index and $\left\{{\hat{y}}_{j,u},{\hat{y}}_{j,l}\right\}$ are the *y* coordinates of the two splines at column *j*.

The reason why it is expected that relation (6) provides a good estimation of the diameter is that in these images the artery wall is almost perfectly horizontal, especially in case an ROI is selected. Indeed, the correctness of this assumption was confirmed a posteriori by the validation of the method, showing a very good correlation among the values of the diameters and FMD with those of the gold standard.

The other parameters which characterize the FMD function are (i) the time interval between cuff deflation and peak diameter, (*time to peak diameter*) and (ii) the FMD percent diameter dilation, given by the percent difference of the peak diameter with respect to the baseline:

(7) $$\text{FMD}=\frac{d_{\text{peak}}-d_{\text{basal}}}{d_{\text{basal}}}[\%].$$

The value of the diameter as a function of the frame position within the movie (or, equivalently, as a function of time) is stored for further analysis. Following the request of the cardiologist, a smoothed version of the diameter plot is also made available as an option. This is obtained by low-pass filtering of the original profile with a kernel of length three.

## 3. RESULTS AND DISCUSSION

The gold standard for validation was obtained by manual segmentation of the artery by an expert.

The criteria used for performance assessment are *accuracy, reproducibility,* and *repeatability* of the measurements. It is worth to outline that the only source of variation in the performance of the algorithm is due to the human intervention, namely, the manual positioning of the ticks setting the units, the choice of the observation window, and the patient movement. The first one introduces a systematic scaling in the measurements expressed in metric units (micron), while leaves unchanged the ones expressed as numbers of pixels. This also applies to the comparison of the measured values with those obtained by the gold standard. Accordingly, the system validation presented hereafter refers to repeatability and reproducibility in such respect.

Concerning the preprocessing, the only step that could compromise the accuracy of the measurements is downsampling. In order to assess this, we performed the diameter measurements on a typical video sequence and compared the results obtained without subsampling. The ticks were fixed on the full-resolution reference image and kept in the same position after downsampling to avoid the systematic error mentioned above. The full images were used (i.e., the ROI functionality was disabled).

The correlation coefficient between the two sets of measurement of the diameter was 0.99841, and the one-way ANOVA analysis confirmed that the two sets of samples are obtained from two distributions having the same mean (*P* = .57). The signal-to-noise ratio (SNR) between the two curves is 62.77 dB. For the FMD, according to relation (7), the accuracy depends on that of the diameter estimation as well as on the positioning of the peak. Tests performed on a set of typical sequences revealed that downsampling leads to a change in the FMD in the range 0.1–0.3%, which is smaller than the variation due to the other causes mentioned above. Accordingly, switching on the downsampling option would not compromise the accuracy of the measurements.

In what follows, the setup used for validation is described.

- *Accuracy:* 100 ultrasound images of an artery were analyzed manually using a modified version of Image  J [16]. The average of two such measurements was considered as the *gold standard*.
- *Repeatability:* 25 healthy young volunteers (age range 24–37, 9 females) underwent measurement of arterial diameter and FMD twice with a delay of 24 hours.
- *Reproducibility in FMD studies:* 65 ultrasound images of the artery and 65 complete FMD movies were analyzed twice using our software. The movies were acquired in 27 healthy volunteers (age 23 to 30 years, 12 males), 12 hypertensive patients (age 40–60, 6 males), 8 patients with coronary artery disease (age 45–65, all males), 8 smokers (age 28–30, 5 females), and 10 patients with congestive heart failure (age 60–78, 7 males).
- *Reproducibility in IMT studies:* 60 US images of the posterior wall of the common carotid artery were analyzed twice using our software. The images were acquired in 15 healthy volunteers (age 23 to 30 years, 7 males) and 45 patients with coronary artery disease (age 45–65, 40 males).

Healthy volunteers included medical residents with negative anamnesis of any active disease, nonsmokers, with a systolic blood pressure lower than 130 mmHg, and a diastolic blood pressure lower that 80 mmHg. Smokers had a history of 5–10 cigarettes/day for 3–10 years and were asked to smoke one cigarette immediately before FMD measurement. Hypertensive subjects (age 40–60, 6 males) had undergone 24-hour blood pressure monitoring which documented average systolic values larger than 140 and diastolic values larger than 90 mmHg. FMD was measured before initiation of antihypertensive therapy. The diagnosis of coronary artery disease was made based on coronary angiography. These patients were on treatment with aspirin. All other active treatments were suspended for at least 24 hours. Congestive heart failure patients included subjects with symptoms of heart failure (NYHA class II-IV) and an ejection fraction lower than 40%.

### 3.1. Performance analysis

To characterize the system performance, the following descriptors were used.

- Intraclass correlation coefficient (*ICC*);
- coefficient of variation (*CV*). It is a measure of the dispersion of a probability distribution and it is calculated as the standard deviation (SD) of repeated measurements divided by their mean:
  (8) $$CV=100\times \frac{\sigma }{\mu },$$
  where *σ* and *μ* are the standard deviation and the mean of the measurement distribution, respectively;
- range of variation (*R*). It is calculated for repeatability and reproducibility studies as the mean of the absolute differences between repeated measurements;
- bias (*B*) across separate measurements. Bias is given by the mean of these differences.

Bland and Altman plots [23] were derived. In all cases, analysis was performed in a randomized, blinded fashion. Statistical analysis for the reproducibility studies was performed as recommended by published guidelines [24] using Statview (SAS Institute, NC, USA). FMD was calculated as in (7). Besides the patient movement, there are two potential causes of variability in the measurements. The first one is the positioning of the observation window (ROI), and the second is the manual positioning of the tags setting the units. The following sections provide the characterization of the system.

#### 3.1.1. Accuracy

**Figure 5:**
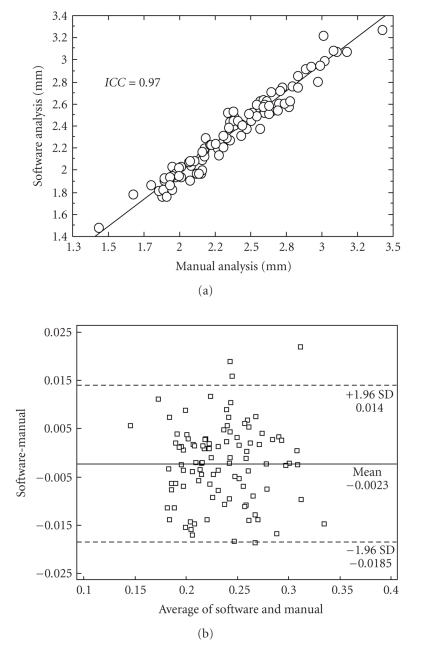
FMD analysis. (a) Correlation between manual (gold standard, horizontal axis) and automatic (vertical axis) analyses; (b) Bland and Altman plot: difference between software and manual measurements versus their average.

In order to assess the accuracy, 100 frames were analyzed both manually and using the new software. The two sets of measurements are highly correlated, as illustrated in Figure 5(a). The intraclass coefficient was *ICC* = 0.97 (*P* < .0001). The range of variation was equal to *RV* = 76 (2–220) *μ*m for arteries with an average diameter of 2.35 mm. The Bland and Altman plot is shown in Figure 5(b).

#### 3.1.2. Repeatability

**Figure 6:**
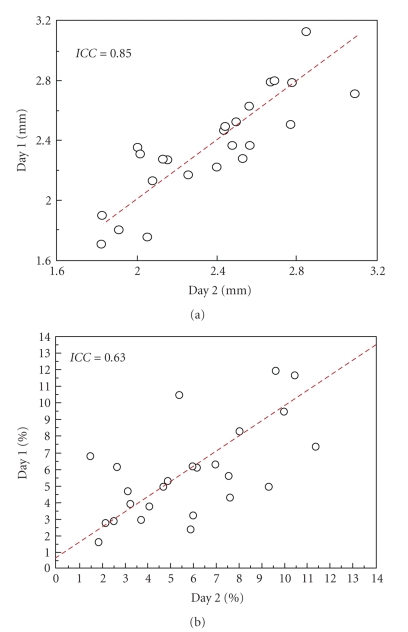
Correlation between repeated measurements in 25 healthy subjects studied twice with a delay of 24 hours. (a) Resting diameter; (b) FMD. The interclass correlation coefficients are *ICC* = 0.85 and *ICC* = 0.63, respectively.

The intraclass coefficient for diameter measurements repeated with a delay of 24 hours on the same 25 subjects was *ICC* = 0.85 (Figure 6(a)). The intraclass correlation coefficient for repeated FMD measurements following the same paradigm was *ICC* = 0.63 (Figure 6(b)). The *RV* for each of these variables was 0.15 (0.01–0.40) mm for the diameter and 1.7 (0.1–5.2)% for FMD, respectively. *CV* was 4.5% for the diameter measurements.

#### 3.1.3. Reproducibility

**Figure 7:**
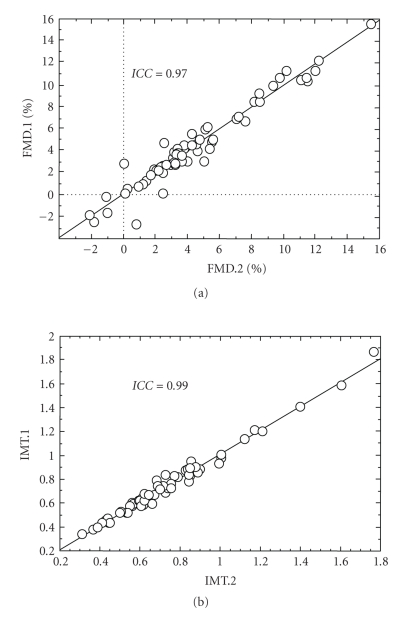
Repeatability. (a) FMD; (b) IMT.

**Figure 8:**
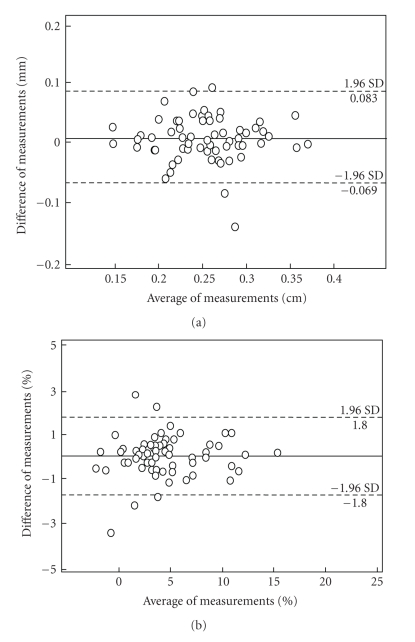
Bland and Altman plots of repeated measurements. The horizontal axis reports the average of two consecutive measurements, the vertical axis their difference. The dotted lines represent 1.96 standard deviation from the mean. (a) Diameter; (b) FMD.

- *Diameter:* 65 frames were analyzed twice using the software. The mean arterial diameter was 2.55 ± 0.05 mm and 2.54 ± 0.05 mm, respectively. The intraclass coefficient across the two measurements was ICC = 0.998 (*P* < .0001). The coefficient of variation for repeated measures of arterial diameter was 0.8 (0.0–3.5)%, and the mean range of variation between the two sets of measurements was 28 (0–143) *μ*m. The bias across the two sets of measurements was *B* = 7 *μ*m with a standard deviation of the differences of 39 *μ*m. The Bland and Altman plot is presented in Figure 8(a).
- *FMD:* when 65 FMD studies were analyzed twice, the intraclass coefficient was *ICC* = 0.969 (*P* < .0001), and the mean range of variation between the two sets of measurements was *RV* = 0.6 (0.2–3.5)% (Figure 7(a)). The bias across the two sets of measurements was 0.3% with a standard deviation of the differences of 0.9%. The Bland and Altman plot is presented in Figure 8(b). When all subjects were considered, the coefficient of variation for FMD was 16%. The coefficient of variation in healthy volunteers (FMD = 6.0 ± 0.8%) was 8.3%. FMD was significantly higher in healthy volunteers compared to the other groups (smokers: 4.9 ± 1.9; hypertensive: 4.5 ± 0.7%; coronary artery disease: 1.7 ± 0.6%; heart failure: 2.9 ± 1.4%, *P* < .05 among groups).
- *IMT:* sixty IMT images were analyzed twice. The intraclass coefficient for these measurements was *ICC* = 0.99, (*P* < .0001, Figure 7(b)), and the mean range of variation between the two sets of measurements was 0.03 mm; the bias across the two sets of measurements was 0.017 mm with the SD of the differences 0.037 mm. The coefficient of variation was 3.6%. These parameters of reproducibility compare favorably with those of previously published methods [20]. IMT was significantly lower in healthy volunteers compared to coronary artery disease patients (coronary artery disease: 0.8370.27 mm; healthy: 0.5060.11 mm, *P* < .00001).

## 4. SUMMARY AND CONCLUSIONS

We describe a simple, accurate, and highly reproducible edge-detection wall-tracking software for the analysis of peripheral endothelial function and IMT. As compared to manual and semiautomatic analyses [16], the proposed software limits significantly measurement errors, reducing the sample size necessary for studies and increasing the reliability of FMD measurements in clinical practice. As well, as compared to previously presented software with the same functions, the one presented here is more rapid and allows manual correction at any point during the automatic analysis. In particular, the user friendliness and rapidity of the analysis (an average of 60 seconds for a full analysis as compared to other software requiring up to 10 minutes [12]) are particularly important characteristics when planning to use this type of analysis in large population studies. Furthermore, the software presented here has the unique advantage that it allows saving both numerical results and a movie containing the echo-tracking analysis. This provides researchers with the possibility of auditing results at any time, which in our experience improves significantly the quality of the data and facilitates interinstitution cooperation. Because in our system image acquisition and analysis are digital, picture quality and resolution are preserved as compared to other systems that require transfer of images from digital to analog and back to digital.

Other features of clinical relevance are as follows.

- Continuous analysis of FMD movies. The proposed system enables the continuous analysis of FMD movies. In the considered setup, the duration of a typical FMD movie is 10 minutes: 1 minute baseline diameter recording, 4.5 minutes cuff inflation, and 4.5 minutes cuff deflation. The software presented here performs arterial diameter measurement throughout these 10 minutes in order to construct a continuous FMD curve. Studies have shown that, as compared to healthy subjects, the FMD response in patients with cardiovascular disease is both lower and delayed in time [25]. Therefore, this time shift should be considered an important component in the characterization of the endothelial dysfunction and the importance of the detection of true peak responses (and of the measurement of the time delay between cuff deflation and peak diameter) should be emphasized. The software proposed here calculates the FMD, the time lag, and the slope between cuff deflation and peak arterial diameter, providing both spatial and temporal parameters characterizing the endothelial function.
- Improved reproducibility. Previous reproducibility studies have reported that observer error accounts for as much as 60% of the within subject variation for both FMD and IMT measurements [6]. As compared to manual analysis, automatic edge-detection is not affected by interobserver variability, which allows a substantial reduction in the number of subjects necessary for clinical studies under the assumption that this is the sole factor limiting the reproducibility. However, it has to be recognized that US resolution, artefacts, and patient movements are the major sources of variation in FMD studies. The range of variation for repeated measures performed using modern software, including the one presented here, approximates the maximum reachable with currently available US technologies. Use of a stereotactic clamp with micrometric movements for the ultrasound probe, ECG triggering as well as optimal immobilization of the arm, a quiet and temperature-controlled environment, and a fasting state remain of great importance. In our laboratory, the radial artery is preferred over the brachial because it can be more solidly immobilized, minimizing movement artefacts. While having a comparable FMD (6-7% in healthy volunteers), the smaller diameter of the radial artery represents a challenge for the type of software presented here.
- Online versus offline analyses. Previously published software allows online image analysis of vessel diameter, which has the advantage of reducing study times [11]. In contrast, our offline technique requires vessel images to be stored digitally for later analysis. While this prolongs analysis time (by 60–90 seconds in average), the advantage of offline analysis is portability (e.g., stored images can be analyzed at any time in randomized batches). As well, an online analysis system implies that the region of interest is selected at the beginning of the study, which is limited by the unpredictability of artifacts. The first window of our software gives a reference frame obtained from the whole 10 minutes of registration, allowing to choose the most artefact-free region of interest. Furthermore, our software is designed to allow interrupting and editing the analysis (including changing the ROI) in the case of patient movement. This source of artifacts is an important limitation of online and previous offline analysis methods [26]. The possibility of such “interactivity” dramatically improves the robustness (i.e., the ability to provide results without failures due to movement artifacts) of the analysis as recently shown in [11]. Both online and offline systems have relative advantages and disadvantages. Our opinion is that a rapid offline analysis that allows operator interaction is an alternative to online analysis facilitating and speeding up both data collection and analysis.
  Overall, we believe that the proposed solution will trigger new interest in the field because of its low cost, portability, interactivity, and user friendliness.

## Figures and Tables

**Figure 9:**
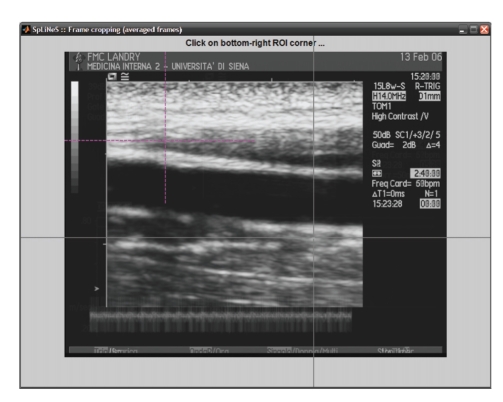
Reference frame: ROI selection.

**Figure 10:**
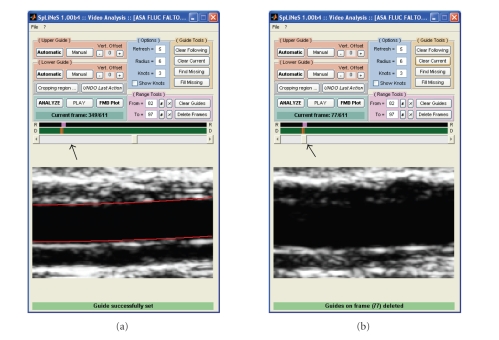
The first window of the software presents an “average frame” derived as an average of all frames in the video and is used to manually set the ROI. The second window requires the operator to click on two calibration marks to calibrate the images.

**Figure 11:**
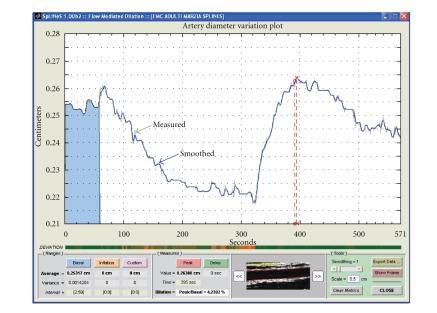
FMD data. Thin line: artery diameter as a function of frame index. Thick line: artery diameter after smoothing. Red line: frame selection.

## APPENDIX

The system is equipped with a GUI that has been designed following the recommendations of the cardiologist of our team. It allows a high degree of interactivity at any time during the processing, which is a must when analyzing low-quality images and videos. To initialize the procedure, the user is asked to click on two positions close to the artery wall in a prototype frame used as reference, that has been previously obtained by averaging all frames in the video. This allows selecting an ideal crop area, where the image will remain stable and the arterial contours sharp throughout the FMD study (see Figure 9) in case the ROI option are activated. A second window allows the operator to click on two contiguous calibration marks in the image in order to set the number of pixels corresponding to a known distance (in [cm]), thus providing quantitative measurement of the arterial diameter in metric units.

The human intervention is possible at any time during the analysis through the manual displacement of the knots in case either the position of the contour in the current frame is judged unsatisfactory or the quality of the current frame is so poor that the algorithm would not converge. In this case, the doctor can also decide to remove the concerned group of frames without affecting the automatic analysis in the rest of the movie.

Figure 10 gives an example. A color bar (indicated as D) gives an index of how parallel the splines remain throughout the study thus facilitating the detection of artifacts: a red color corresponds to a movie section of worse quality. Positioning the cursor in correspondence of these frames enables manual analysis, for their eventual removal. Finally, the FMD data are presented as a plot as illustrated in Figure 11. The GUI allows the evaluation of the set of measurements and the visualization of the frame corresponding to a given diameter. The tract of artery analyzed is typically 0.5–1 cm long. Intervals for each relevant condition (baseline, peak dilation) can be set. In order to facilitate the recognition of outliers, a frame preview window is added to this window.
